# The Anti-Tumor Effect and Underlying Apoptotic Mechanism of Ginsenoside Rk1 and Rg5 in Human Liver Cancer Cells

**DOI:** 10.3390/molecules26133926

**Published:** 2021-06-27

**Authors:** Chen Chen, Qing Lv, Yang Li, Ying-Hua Jin

**Affiliations:** Key Laboratory for Molecular Enzymology and Engineering of the Ministry of Education, School of Life Sciences, Jilin University, Changchun 130012, China; cchen16@mails.jlu.edu.cn (C.C.); lvqing@gensci-china.com (Q.L.)

**Keywords:** ginsenoside, human liver cancer, cytotoxicity, network pharmacology, bioinformatics analysis, endogenous apoptotic pathway

## Abstract

Ginsenoside Rk1 and Rg5 are minor ginseng saponins that have received more attention recently because of their high oral bioavailability. Each of them can effectively inhibit the survival and proliferation of human liver cancer cells, but the underlying mechanism remains largely unknown. Network pharmacology and bioinformatics analysis demonstrated that G-Rk1 and G-Rg5 yielded 142 potential targets, and shared 44 putative targets associated with hepatocellular carcinoma. Enrichment analysis of the overlapped genes showed that G-Rk1 and G-Rg5 may induce apoptosis of liver cancer cells through inhibition of mitogen-activated protein kinase (MAPK) and nuclear factor-kappa B (NF-κB) signal pathways. Methyl thiazolyl tetrazolium (MTT) assay was used to confirm the inhibition of cell viability with G-Rk1 or G-Rg5 in highly metastatic human cancer MHCC-97H cells. We evaluated the apoptosis of MHCC-97H cells by using flow cytometry and 4′,6-diamidino-2-phenylindole (DAPI) staining. The translocation of Bax/Bak led to the depolarization of mitochondrial membrane potential and release of cytochrome *c* and Smac. A sequential activation of caspase-9 and caspase-3 and the cleavage of poly(ADP-ribose) polymerase (PARP) were observed after that. The levels of anti-apoptotic proteins were decreased after treatment of G-Rk1 or G-Rg5 in MHCC-97H cells. Taken together, G-Rk1 and G-Rg5 promoted the endogenous apoptotic pathway in MHCC-97H cells by targeting and regulating some critical liver cancer related genes that are involved in the signal pathways associated with cell survival and proliferation.

## 1. Introduction

**Figure 1:**
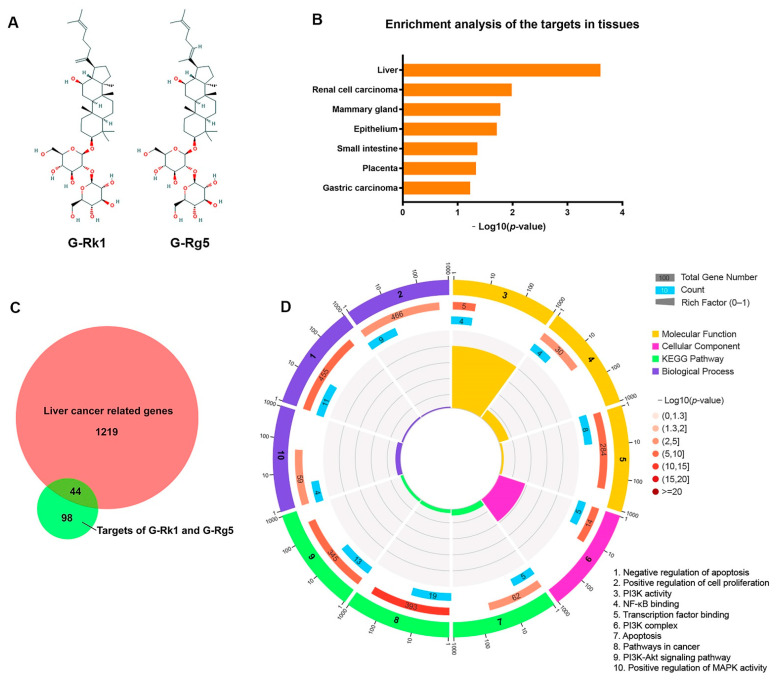
G-Rk1 and G-Rg5 targeting hepatocellular carcinoma related genes with an anti-cancer effect. (**A**) Chemical structures of G-Rk1 and G-Rg5. (**B**) Enrichment analysis of the potential target genes of G-Rk1 and G-Rg5 in human tissues. (**C**) The Venn diagram showed the crosstalk between hepatocellular carcinoma related genes and targets of two ginsenosides. The red part indicates hepatocellular carcinoma related genes, and the green part indicates targets of G-Rk1 and G-Rg5. (**D**) Gene Ontology (GO) and KEGG pathway enrichment analysis of the overlapped genes.

Panax ginseng, a traditional Chinese medicinal herb, is derived from the roots of Araliaceae plants [1]. It has been widely used for treatment of various diseases in Asia for nearly 5000 years [2]. Ginsenosides, a class of triterpenoid saponins, are secondary metabolites extracted from ginseng [3]. More than 150 naturally occurring ginsenosides have been identified to date, which are classified into two major groups: oleanane and dammarane. Among them, dammarane type ginsenosides can be divided into protopanaxadiol saponins (PPD) and protopanaxatriol saponins (PPT) [3,4]. The content of total saponins in the roots of Panax ginseng is about 4–8%. The major saponins including Rb1, Rb2, Rc, Rd, Re, Rg1, and Rf, account for 80–90% of total saponins [3]. Through the in-depth study on biotransformation, separation and purification, pharmacological activity, and action mechanisms of ginsenosides, it is shown that the major natural ginsenosides with high content in ginseng have low bioavailability because of their poor oral absorption in the human body [5]. It is reported that the oral bioavailability of protopanaxadiol ginsenosides (including Rb1, Rb2, and Rd) and protopanaxatriol ginsenosides (including Rg1, Re, and Rf) are less than 5% [6]. Red ginseng is a processed product of Panax ginseng by steaming and drying the raw ginseng. Many ginsenosides are found in red ginseng, but not in raw ginseng, because the major ginsenosides need to be transformed into the minor ginsenosides through a deglycosylation [7]. The sugar moieties at carbon-20 of ginsenosides Rb1, Rc, and Rd were deglycosylated by heat-processing and then gradually changed into (20*S*)G-Rg3, (20*R*)G-Rg3, G-Rg5, and G-Rk1. Ginsenoside Rk1 and Rg5 are positional isomers of each other depending on the position of the double bonds at carbon-20(21) or -20(22) (Figure 1A). Additionally, the formation of this double bond is caused by the elimination of H_2_O at carbon-20 of Rg3 by high pressure and temperature [7,8]. Studies have shown that these minor ginsenosides transformed from major ginsenosides have higher bioavailability because of their simpler glycosyl groups. Moreover, Ginsenoside Rk1 and Rg5 have been confirmed to have many pharmacological effects, for example, anti-inflammatory activity, anti-tumor effect, nervous system protection, memory improvement, etc. [9,10,11].

Liver cancer, an aggressive tumor which frequently occurs in chronic liver disease and cirrhosis, is the third leading cause of cancer-related death in the world [12]. The occurrence and development of liver cancer is a complex network process with participation of multiple genes, which are involved in lots of pathways, such as extracellular matrix degradation and adhesion to promote tumor metastasis and invasion, angiogenesis to support tumor metabolism, inhibition of cell cycle arrest, and apoptosis to promote the survival and proliferation of cancer cells [13,14,15,16]. Globally, the highest liver cancer rates are found in East and Southeast Asia and Middle and Western Africa, which reflect geographic prevalence of viral hepatitis and environmental pathogens [17]. In addition to hepatitis virus, the inducing factors of liver cancer include alcoholism, obesity, diabetes and heredity [18]. At present, chemotherapy and radiotherapy are still the main systemic therapies in treatment of liver cancer, together with immunotherapy developed in recent decades [19,20]. However, these therapies are usually accompanied with drug resistance, which leads to poor prognosis of patients [21]. Therefore, it is imperative to develop a new type of adjuvant drug for liver cancer therapy.

Apoptosis is an evolutionarily conserved form of programmed cell death involving a family of caspase proteases [22]. The previous studies showed that cell apoptosis can be divided into an endogenous pathway and exogenous pathway according to different caspase activation cascades [23]. The mitochondrial pathway is mediated by the translocation of Bax/Bak to mitochondrial outer membrane and the subsequent release of cytochrome *c*, leading to the activation of caspase-9. Another exogenous pathway is triggered by the oligomerization of cell-surface death receptors and the following activation of caspase-8 [24,25]. Deficiency in apoptosis may lead to tumor progression and drug resistance, and many anti-cancer drugs that promote apoptosis have already been used in clinical therapy.

Although, previous studies have reported the cytotoxic activity of G-Rk1 and G-Rg5 in various cancer cells, the underlying mechanism was not elucidated. Our current study aimed to evaluate whether G-Rk1 or G-Rg5 induce apoptosis in highly metastatic human liver cancer MHCC-97H cells and to investigate the underlying mechanism.

## 2. Results

### 2.1. G-Rk1 and G-Rg5 May Serve as Potential Drugs for Treatment of Hepatocellular Carcinoma by Targeting Its Related Genes

Previous studies confirmed the anti-cancer effects of G-Rk1 and G-Rg5 on hepatocellular carcinoma [26,27]. However, the advantages of these two ginsenosides in treatment of liver cancer and the specific anti-tumor mechanisms still need to be clarified. Swiss Target Prediction, Similarity Ensemble Approach (SEA), and TargetNet were used to predict the targets of G-Rk1 and G-Rg5. There were 119 genes targeted by G-Rk1 and 97 genes targeted by G-Rg5. Our results showed that G-Rk1 and G-Rg5 yielded 142 potential targets after eliminating all duplicates. Then, we performed enrichment analysis of these 142 potential targets in tissues using DAVID. We found these genes were significantly enriched in liver (Figure 1B), indicating that G-Rk1 and G-Rg5 may play an important role in treatment of liver disease. To further determine the pharmacological mechanisms of G-Rk1 and G-Rg5 against liver cancer, the related genes of hepatocellular carcinoma were obtained from GeneCards database [28]. The Venn diagram analysis showed that G-Rk1 and G-Rg5 shared 44 putative targets with the genes in hepatocellular carcinoma dataset (Figure 1C and Table S1). Next, we wanted to know the possible functions of the overlapped genes, and performed GO and KEGG pathway enrichment analysis of these 44 genes by setting all human genes as background. The top enrichments in molecular function, cellular component, biological process and KEGG pathway categories were some functions related to cell proliferation and apoptosis (Figure 1D and Figure S1). As we know, PI3K-Akt, NF-κB and MAPK are all pivotal signals involved in a wide variety of cellular processes such as proliferation, differentiation, and transcriptional regulation. Taken together, we speculated that G-Rk1 and G-Rg5 may inhibit the survival and proliferation of cancer cells and induce apoptosis by targeting several hepatocellular carcinoma related genes so as to exert their anti-tumor effect.

### 2.2. G-Rk1 or G-Rg5 Inhibited the Survival of MHCC-97H Cells and Induced Apoptotic Cell Death

**Figure 2:**
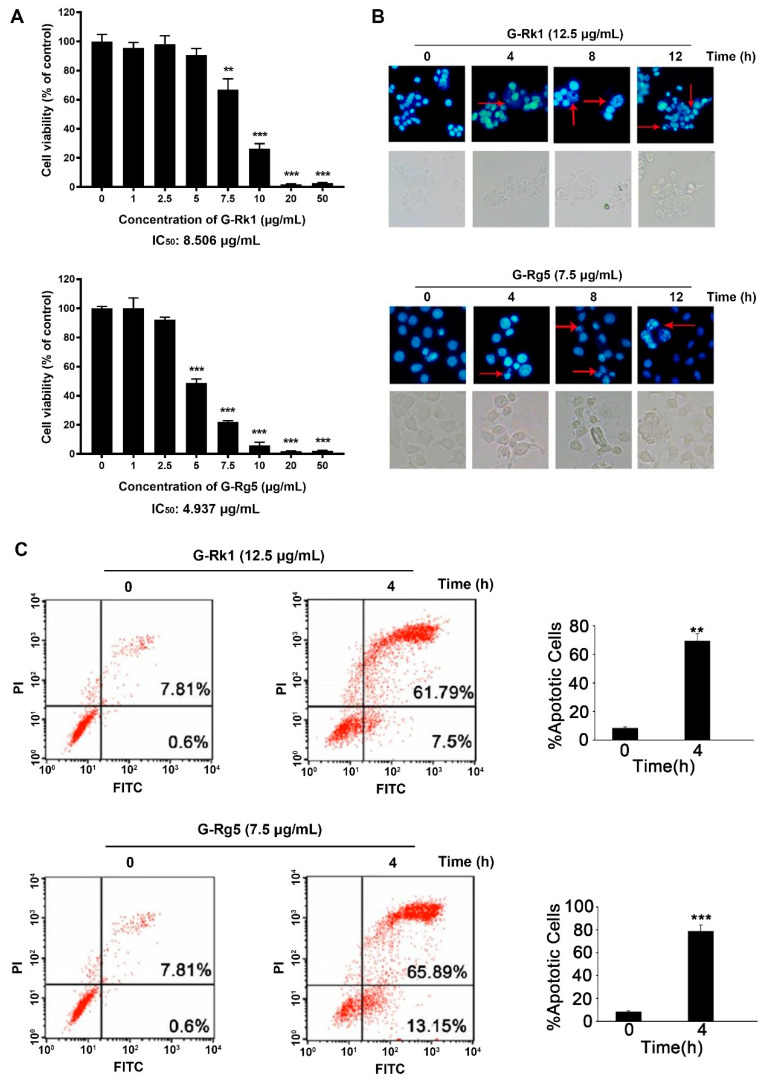
Cytotoxic effect of G-Rk1 and G-Rg5 on MHCC-97H cells. (**A**) MHCC-97H cells were treated with various concentrations of G-Rk1 or G-Rg5 for 48 h. Cell viability was determined by MTT assay. (**B**) MHCC-97H cells were treated with 12.5 μg/mL G-Rk1 or 7.5 μg/mL G-Rg5 for the indicated times. The cells were stained with DAPI to visualize the morphological changes under a fluorescence microscope. The red arrows indicate chromatin condensation. (**C**) MHCC-97H cells were treated with G-Rk1 or G-Rg5 for 0 h or 4 h. The cells were stained with Annexin V-FITC/PI, and the degree of apoptosis was determined by measuring the population of Annexin V-FITC/PI-positive cells by flow cytometry. All data are shown as the mean ± SD of experiments performed in triplicate. A Student’s *t*-test was used for quantitative analysis with *** presenting *p* < 0.001 and ** presenting *p* < 0.01.

To examine whether G-Rk1 or G-Rg5 can inhibit the proliferation of human liver cancer MHCC-97H cells, we assessed the viability of MHCC-97H cells after treatment with G-Rk1 or G-Rg5 using MTT assay. It was observed that G-Rk1 or G-Rg5 strongly inhibited cell growth in MHCC-97H cells (Figure 2A). A significant reduction in cell viability indicated that both of the G-Rk1 and G-Rg5 have a dose-dependent cytotoxicity against MHCC-97H cells, with an IC_50_ value about 8.506 μg/mL and 4.937 μg/mL, respectively. Additionally, in subsequent experiments, we chose a 1.5-fold of IC_50_ concentration in a time-dependent manner. Then, we determined the morphological changes in G-Rk1/G-Rg5 treated MHCC-97H cells. After treatment with G-Rk1/G-Rg5 for 12 h, MHCC-97H cells showed the typical morphological characteristic of apoptosis: obviously cell rounding and membrane blebbing (Figure 2B). DAPI staining showed that G-Rk1 or G-Rg5 also induced nuclear fragmentation and chromatin condensation in a time-dependent manner (Figure 2B). The fraction of Annexin V-FITC/PI-positive cells were detected to characterize early and late apoptotic cell death by flow cytometry. The result showed that over 7.5% early apoptosis and 61.79% late apoptosis appeared after treatment with 12.5 μg/mL G-Rk1 for 4 h. Meanwhile, the numbers of early and late MHCC-97H apoptotic cells were 13.15% and 65.89%, respectively, after treatment with 7.5 μg/mL G-Rg5 for 4 h (Figure 2C). In conclusion, G-Rk1 and G-Rg5 inhibited the survival and proliferation of MHCC-97H cells by inducing apoptosis.

### 2.3. G-Rk1 or G-Rg5 Induced the Apoptosis of MHCC-97H Cells through the Release of Cytochrome c and Smac

**Figure 3:**
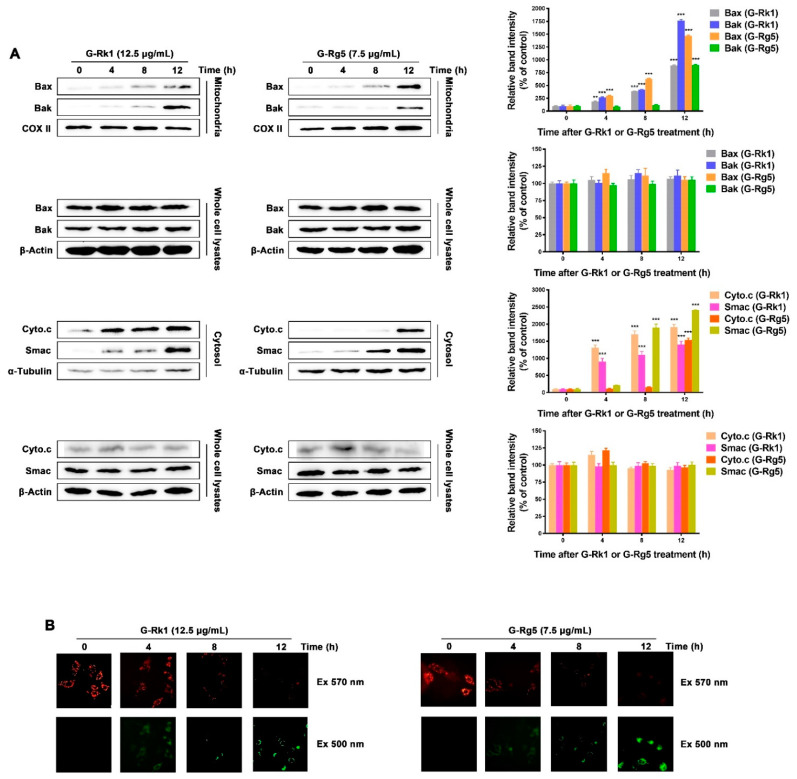
G-Rk1 or G-Rg5 induced apoptosis of MHCC-97H cells is mediated through Bax/Bak translocation and release of cytochrome *c*/Smac. (**A**) G-Rk1 or G-Rg5 induced Bax/Bak translocation from cytoplasm to mitochondrial outer-membrane and release of cytochrome *c*/Smac were analyzed by immunoblotting for the indicated times. The right panel is quantitative analysis of Western blot. Data are shown as the mean ± SD of experiments performed in triplicate. A Student’s *t*-test was used for quantitative analysis with *** presenting *p* < 0.001 and ** presenting *p* < 0.01. (**B**) MHCC-97H cells were treated with 12.5 μg/mL G-Rk1 or 7.5 μg/mL G-Rg5 for the indicated times and stained with MitoCapture cation dye. The same fields of cells were visualized with excitation wavelengths of 570 nm and 500 nm, respectively.

In order to tell whether G-Rk1 or G-Rg5 induced apoptosis in MHCC-97H cells through the mitochondrial pathway, we performed mitochondrial membrane potential depolarization assay. MHCC-97H cells were treated with 12.5 μg/mL G-Rk1 or 7.5 μg/mL G-Rg5 in a time-dependent manner, and stained with the mitochondria-specific cation dye MitoCapture. The dissipation of mitochondrial membrane potential in treatment with G-Rk1 or G-Rg5 was coincident with the increased level of cytochrome c and Smac in cytoplasm (Figure 3A,B). At the same time, under the treatment of G-Rk1 or G-Rg5, the expression levels of cytochrome c, Smac, Bax, and Bak remained almost constant in whole cell lysates (Figure 3A). Additionally, the increase of Bax and Bak in mitochondria indicated their translocation from cytosol to mitochondrial membrane. Pro-apoptotic proteins such as cytochrome c and Smac were subsequently released from mitochondria to cytosol through the opening pores formed by Bax and Bak (Figure 3A). The above data demonstrated that G-Rk1 or G-Rg5 induced apoptosis in MHCC-97H cells was mediated by the translocation of Bax/Bak and release of cytochrome c/Smac.

### 2.4. G-Rk1 or G-Rg5 Induced Apoptosis in MHCC-97H Cells by Activating Caspase-9 and Decreasing the Levels of Anti-Apoptotic Proteins

**Figure 4:**
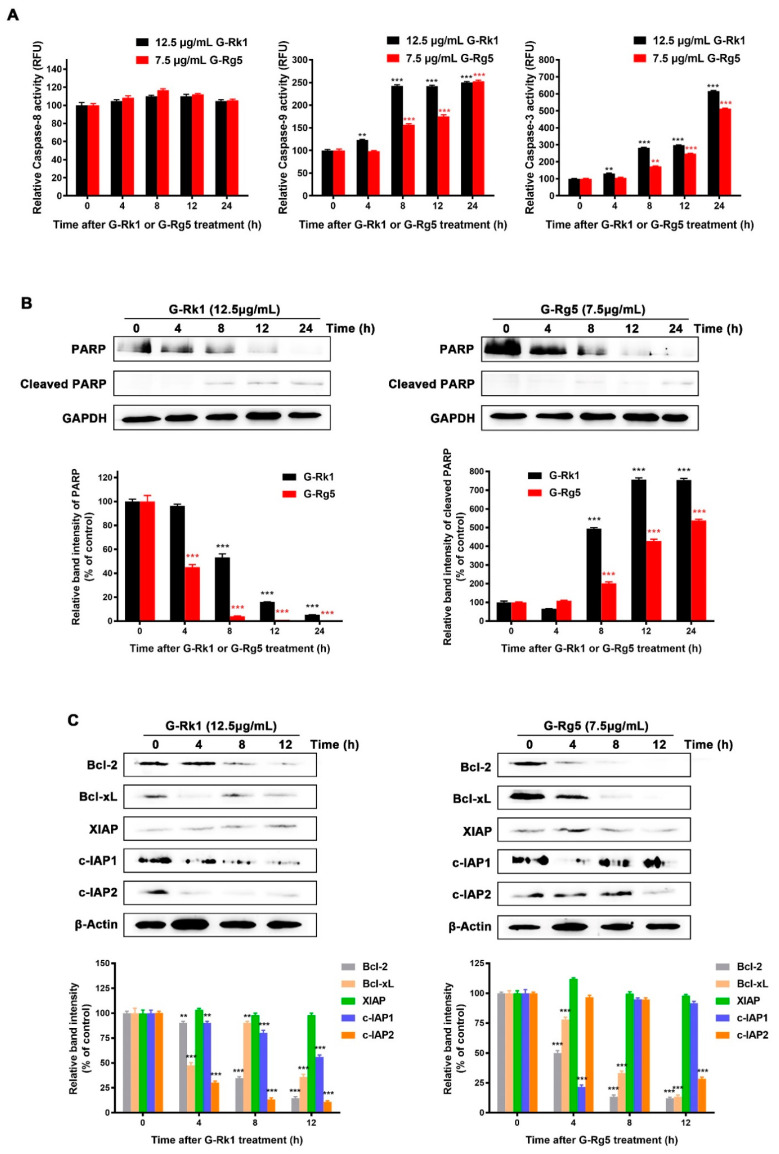
Activation of caspase-8, -9, -3 and analysis of anti-apoptotic protein levels in MHCC-97H cells treated with G-Rk1 or G-Rg5. (**A**) MHCC-97H cells were treated with 12.5 μg/mL G-Rk1 or 7.5 μg/mL G-Rg5 for the indicated times. Cell-free caspase-8, -9, -3 activities were analyzed using specific substrates, Ac-IETD-AFC, Ac-LEHD-AFC, and Ac-DEVD-AFC, respectively. (**B**) Immunoblotting analysis of the caspase-3 substrate PARP cleavage in MHCC-97H cells treated with G-Rk1 or G-Rg5 for the indicated times. (**C**) Immunoblotting analysis of the anti-apoptotic protein levels in MHCC-97H cells. The lower panels are quantitative analyses of the above data in (**B**,**C**). Data are shown as the mean ± SD of experiments performed in triplicate. A Student’s *t*-test was used for quantitative analysis, and the significance is shown as *** *p* < 0.001 and ** *p* < 0.01.

As the above results showed, MHCC-97H cells treated with G-Rk1 or G-Rg5 led to release of cytochrome *c* from mitochondria to cytoplasm, a typical signal transduction in endogenous apoptotic pathway. Then, we investigated the activation kinetics of the initiator caspase-8 and caspase-9, and their downstream effector caspase-3. As shown in Figure 4A, the proteolytic activation of caspase-9 was significantly up-regulated in a time-dependent manner when treated with G-Rk1 or G-Rg5, followed by a gradual elevation of caspase-3 activity, whereas the caspase-8 activity remained not obviously changed. Poly(ADP-ribose) polymerase (PARP) is a specific substrate of the activated caspase-3 [29]. The immunoblotting analysis showed that the activated caspase-3 cleaved PARP to yield a 85 kDa fragment after treatment with G-Rk1 or G-Rg5 in a time-dependent manner (Figure 4B). These results demonstrated that the apoptosis of MHCC-97H cells induced by G-Rk1 or G-Rg5 was mediated by caspase-9, but not caspase-8 through an endogenous apoptotic pathway. Next, we also measured the levels of anti-apoptotic proteins to further understand the mechanisms of G-Rk1/G-Rg5-induced cell apoptosis. We found that the levels of anti-apoptotic proteins decreased in varying degrees, among which Bcl2, Bcl-xL, and c-IAP2 were relatively obviously down-regulated after treatment with G-Rk1 or G-Rg5 (Figure 4C).

## 3. Discussion

Thus far, a large number of natural compounds have been identified to have anti-tumor effects. The action mechanisms of these compounds are varied, including inducement of endoplasmic reticulum stress and cell cycle arrest, inhibition of ATPase and protein synthesis, and promotion of apoptosis [30,31,32,33,34]. Ginsenosides, the main active ingredients in ginseng, have been suggested to have therapeutic effects on a variety of human diseases, such as senescence, hyperglycemia, hypertension, hypoimmunity, and cancer. However, the underlying mechanisms of ginsenosides on these diseases remain to be clarified, so it limits their clinical application. Ginsenosides are triterpene saponins with a terpenoid steroidal nucleus connected to glycosyl groups, also known as plant hormones. The structure of ginsenosides indicates that they can competitively bind to some proteins and affect intracellular signal transduction. Our previous studies have reported the identification of ginsenoside targets based on phage display. For example, (20S)G-Rh2 has been identified to inhibit Annexin A2 binding to NF-κB p50 subunit [35]. Subsequently, ginsenoside compound K (CK), G-Rk1, and G-Rg5 were also confirmed to inhibit NF-κB by targeting Annexin A2 [36,37]. There is a phenomenon that the addition of bovine serum albumin (BSA) reduces the cytotoxic effects of compounds on various kinds of cells appearing in the experiments of cell viability. Additionally, BSA is often designed as a carrier for several small molecular drugs to achieve sustained release [38]. It has been reported that human serum albumin (HSA) can interact with (20S)G-Rh2, (20S)G-Rg3, G-Rk1, G-Rg5, G-Rh4, and CK because of the sequence homology between HSA and BSA. Additionally, the binding sites are located in different domains of HSA [39,40]. Taken together, we can summarize some characteristics of ginsenosides. They can target a variety of proteins to regulate the corresponding signal pathways. This is why ginsenosides have therapeutic effects on kinds of human diseases. Ginsenosides as natural compounds, different from some synthetic compounds, have moderate affinity with target proteins. Additionally, the regulation of this interaction is concentration-dependent. Therefore, the dosage of ginsenosides is larger than that of other compounds in treatment of various diseases. In addition, the bioavailability of major ginsenosides is very low. The minor ginsenosides such as G-Rk1 and G-Rg5, which are transformed from the major ginsenosides through a deglycosylation, have higher bioavailability and absorption [41]. In this study, we confirmed the anti-tumor effect of G-Rk1 and G-Rg5 on highly metastatic liver cancer cell line, MHCC-97H. We considered that finding the potential targets of G-Rk1 and G-Rg5 would help to elucidate the underlying mechanism of the anti-tumor effect on liver cancer cells when treated with G-Rk1 or G-Rg5. Since less information is available on crystal structures of target proteins interacted with ginsenosides, we used bioinformatics method based on chemical structural similarities of G-Rk1 and G-Rg5 and collected 142 potential targets in database. Then, we found 44 putative genes shared with hepatocellular carcinoma in GeneCards. Enrichment analysis of the overlapped genes was then performed, and the results showed that G-Rk1 and G-Rg5 mainly target genes involved in NF-κB, MAPK, and PI3K-Akt pathways, which promote the survival and proliferation of cancer cells. Pharmacological network was established to find out the key regulated genes in these pathways. In Figure S1, the size of node indicates its importance in background network. From the ginsenoside-target-pathway network, we observed that EGFR, ESR1, ESR2, HSP90AA1, and RELA are all critical genes that we should focus on, which may reveal the anti-tumor mechanism of G-Rk1 and G-Rg5. Then, we preliminarily verified the interaction between these proteins and G-Rk1/G-Rg5 using molecular docking. We found that ESR2 and HSP90AA1 were likely to interact with G-Rk1 and G-Rg5, and the binding energy released by interaction is more than 4 kcal/mol (Data are not shown). As we know, some of the anti-apoptotic proteins are regulated by NF-κB and estrogen receptors [35,42]. Combined with the analysis of network pharmacology, RELA, ESR1, and ESR2 are all subunits of the above transcription factors which may regulate the process of cancer cell apoptosis. The inhibition of HSP90AA1 may induce protein misfolding, leading to endoplasmic reticulum stress and subsequent cell cycle arrest and cell death. In summary, G-Rk1 and G-Rg5 exert their anti-tumor effect on liver cancer by targeting several proteins. Additionally, our future studies should focus on the interaction verification and molecular function of these critical target genes.

Inducing apoptosis is an important mechanism of most chemotherapeutic agents in treatment of susceptible cancer cells [43,44]. At the same time, how to internalize these drugs effectively by cancer cells and then trigger apoptosis is also an important issue for drug development. The saponin structure of ginsenosides determines their amphiphilic molecular characteristics. Previous reports have shown that saponins could bring about transient pores in the membrane on account of the unique interaction between saponins and membrane components like cholesterol and phospholipids [45,46]. Therefore, we speculate that G-Rk1 and G-Rg5 are also internalized by cancer cells in this manner and interact with their potential intracellular targets to affect a range of cell signal transduction, and ultimately lead to apoptosis. It was also confirmed by cellular thermal shift assay (CETSA) that G-Rk1 and G-Rg5 can indeed bind to intracellular Annexin A2 in our previous study [36]. Herein, we showed that G-Rk1 and G-Rg5 both have cytotoxic effects on MHCC-97H cells by inducing apoptosis. In our previous study, we determined the cell viability of human umbilical vein endothelial cells (HUVEC) in treatment with G-Rk1 and G-Rg5 by MTT, with an IC_50_ of 12.272 μg/mL and 10.182 μg/mL, respectively [39]. Combined with the results of this study, it demonstrated that the cytotoxic effect of G-Rk1 and G-Rg5 on normal tissue cells is less than that on MHCC-97H cells. Further investigation revealed that both G-Rk1 and G-Rg5 induced MHCC-97H cell apoptosis through the activation of caspase-9, but not caspase-8. It is suggested that the apoptosis induced by G-Rk1 or G-Rg5 in MHCC-97H cells is mainly through the endogenous pathway. We observed that the translocation of Bax/Bak led to the depolarization of mitochondrial membrane potential and release of cytochrome *c* to cytoplasm. Then, a sequential activation of caspase-9 and caspase-3 and the cleavage of PARP were observed after these mitochondrial events. Meanwhile, the levels of several anti-apoptotic proteins were decreased in MHCC-97H cells treated with G-Rk1 or G-Rg5 in a time-dependent manner. Taken together, G-Rk1 and G-Rg5 induced strong and rapid apoptosis in MHCC-97H cells, which was executed by involvement of all three major apoptosis regulating protein families including Bcl-2 family, Caspase family, and IAPs family. In view of the above study, we believe that G-Rk1 and G-Rg5 will become promising adjuvant drugs in treatment of liver cancer. Additionally, we have confidence in their practical application.

## 4. Materials and Methods

### 4.1. Cell Lines and Reagents

MHCC-97H cells were obtained from the Liver Cancer Institute, Fudan University, Shanghai, China. Ginsenoside Rk1 and Rg5 were purchased from Yuanye (≥98% HPLC, Shanghai, China), dissolved in 75% alcohol. Caspase substrates Ac-DEVD-AFC, Ac-IETD-AFC, Ac-LEHD-AFC, and MitoCapture reagent were purchased from Calbiochem (La Jolla, CA, USA). Mitochondria Isolation Kit was purchased from Pierce (Rockford, IL, USA). Annexin V-FITC/PI Double Staining Assay Kit was purchased from Beyotime Biotechnology (Shanghai, China). 3-(4,5-dimethylthiazol-2-yl)-2,5-diphenyltetrazolium bromide (MTT), dimethyl sulfoxide (DMSO), 4′,6-diamidino-2-phenylindole (DAPI), and albumin from bovine serum (BSA) were purchased from Sigma-Aldrich (St. Louis, MO, USA). Fetal calf serum was purchased from Biological Industries (Kibbutz Beit-Haemerk, Israel). Dulbecco modified Eagle’s medium (DMEM) was purchased from Gibco BRL (Grand Island, NE, USA). We used the following primary antibodies: rabbit anti-PARP (Paso Robles, CA, USA), mouse anti-XIAP (Santa Cruz), rabbit anti-c-IAP1 (Santa Cruz), rabbit anti-c-IAP2 (Santa Cruz), rabbit anti-Bcl2 (Cell Signaling; Danvers, MA, USA), rabbit anti-Bcl-xL (Cell Signaling), mouse anti-β-actin (Proteintech, Chicago, IL, USA), mouse anti-α-tubulin (Proteintech), mouse anti-GAPDH (Proteintech), mouse anti-COX II (Cell Signaling), rabbit anti-Bax (Santa Cruz), rabbit anti-Bak (Santa Cruz), rabbit anti-Smac (Santa Cruz), and rabbit anti-Cytochrome *c* (Santa Cruz). We used the following secondary antibodies: HRP-conjugated goat anti-mouse IgG (Pierce) and HRP-conjugated goat anti-rabbit IgG (Pierce).

### 4.2. Cell Culture Condition

Human liver cancer MHCC-97H cells were cultured in DMEM containing 10% (*v/v*) heat-inactivated fetal calf serum with 8 mg/mL ampicillin and 10 mg/mL streptomycin at 37 °C in a humidified atmosphere containing 5% CO_2_.

### 4.3. Cell Viability Assay

Exponentially growing MHCC-97H cells were seeded into a 96-well plate at 1 × 10^4^ cells per well. After incubation for 24 h, the MHCC-97H cells were treated with increasing concentrations of G-Rk1 or G-Rg5 from 0 to 50 μg/mL for 48 h in triplicate. G-Rk1 and G-Rg5 were both diluted in serum-free DMEM. At the end of treatment, 20 μL of MTT (5 mg/mL) was added to each well and the cells were incubated for another 4 h. Then, the culture medium was removed, and the formazan grains formed by viable cells were solubilized with 150 μL DMSO. Absorption at 550 nm of each well was measured using a microplate reader (TECAN, Maennedorf, Switzerland).

### 4.4. DAPI Staining Assay

The MHCC-97H cells were uniformly seeded in a 24-well plate with a cell density of 5 × 10^4^ cells per well and incubated for 24 h. Then, 12.5 μg/mL G-Rk1 or 7.5 μg/mL G-Rg5 diluted in serum-free DMEM was added to each well for 0, 4, 8, and 12 h. The medium was replaced by 4% paraformaldehyde, and the cells were incubated on ice for 3 min. Finally, 300 μL of PBS buffer containing 0.1 μg/mL DAPI was added to each well, and the morphological changes of MHCC-97H cells were observed under a fluorescence microscope.

### 4.5. Annexin V-FITC/PI Double Staining Assay

MHCC-97H cells were uniformly seeded in 100 mm cell culture dishes with a cell density of 1 × 10^6^ cells per dish. Then, the cells were treated with 12.5 μg/mL G-Rk1 or 7.5 μg/mL G-Rg5 in serum-free DMEM for 0 h or 4 h, and stained with Annexin V-FITC/PI reagent. The percentage of Annexin V-FITC/PI-positive cells was determined by flow cytometry (FACSCalibur, Becton-Dickinson, San Jose, CA, USA).

### 4.6. Cell-Free Caspase Activity Assay

MHCC-97H cells were uniformly seeded in 100 mm cell culture dishes with a cell density of 1 × 10^6^ cells per dish. Then, the cells were treated with 12.5 μg/mL G-Rk1 or 7.5 μg/mL G-Rg5 in serum-free DMEM for the indicated times. After treatment, 50 μg of whole cell lysates were incubated with 200 nM Ac-DEVD-AFC (for caspase-3), Ac-IETD-AFC (for caspase-8), and Ac-LEHD-AFC (for caspase-9) in a reaction buffer containing 20 mM HEPES pH 7.4, 100 mM NaCl, 10 mM DTT, 0.1% CHAPS, and 10% sucrose at 37 °C for 1 h. Then, the reaction was monitored by fluorescence at excitation of 405 nm and emission of 535 nm.

### 4.7. Depolarization Assay of Mitochondrial Membrane Potential

MHCC-97H cells were uniformly seeded in a 24-well plate with a cell density of 5 × 10^4^ cells per well. Then, the cells were treated with 12.5 μg/mL G-Rk1 or 7.5 μg/mL G-Rg5 in serum-free DMEM for the indicated times, and incubated with 1 μg/mL MitoCapture cation dye at 37 °C for 30 min. The depolarization of mitochondrial membrane potential was observed and photographed under a fluorescence microscope at excitation of 500 nm and 570 nm, respectively.

### 4.8. Isolation of Mitochondria from Cytosol and Preparation of Protein Extracts

MHCC-97H cells were uniformly seeded in 100 mm cell culture dishes with a cell density of 1 × 10^6^ cells per dish. Then, the cells were treated with 12.5 μg/mL G-Rk1 or 7.5 μg/mL G-Rg5 in serum-free DMEM for the indicated times and harvested. Mitochondrial and cytosolic protein extracts were prepared using a Mitochondria Isolation Kit (Pierce, Rockford, IL, USA) according to the manufacturer’s instructions.

### 4.9. Immunoblotting Analysis

MHCC-97H cells were uniformly seeded in 100 mm cell culture dishes with a cell density of 1 × 10^6^ cells per dish. Then, the cells were treated with 12.5 μg/mL G-Rk1 or 7.5 μg/mL G-Rg5 in serum-free DMEM for the indicated times. The treated cells were harvested and washed with ice-cold PBS and solubilized in RIPA lysis buffer with 1 mM PMSF for 1 h on ice. After centrifugation at 20,000× *g* for 20 min, 50 μg of soluble protein from each sample was analyzed by sodium dodecyl sulfate-polyacrylamide gel electrophoresis, followed by electro-transfer onto a PVDF membrane. Then, the membrane was blocked with 5% non-fat milk in 0.1% PBST and incubated with the indicated antibodies. The blots were washed and incubated with secondary antibodies followed by detection with an electrogenerated chemiluminescence revelation assay.

### 4.10. Establishment of the Ginsenoside-Target-Pathway Interaction Network

Due to the lack of research on ginsenoside targets, we kept all the potential targets in Swiss Target Prediction [47], Similarity Ensemble Approach (SEA) [48], and TargetNet database [49], and eliminated the duplicates. Then, we collected the liver cancer related genes from GeneCards database with relevance score > 20 [28]. We took the intersection of ginsenosides targets and liver cancer related genes, and then constructed the ginsenoside-target-pathway interaction network using Cytoscape software (version 3.6.0).

### 4.11. Gene Ontology and Pathway Enrichment Analysis

The potential target genes of G-Rk1 and G-Rg5 were submitted to DAVID [50], and the functional annotation chart was used for tissue enrichment analysis. The overlapped genes were submitted to DAVID, and the functional annotation clustering was used for GO and KEGG pathway enrichment analysis. Then, the circos diagram was drawn using the OmicShare tools, a free online platform for data analysis (http://www.omicshare.com/tools (accessed on 20 April 2021).

### 4.12. Statistical Analysis

All data were obtained from independent triple-replicated experiment and presented as the mean ± standard deviation (SD). Significance was determined by a two-tail Student’s *t*-test via SPSS v18.0.

## 5. Conclusions

We found G-Rk1 and G-Rg5 shared 44 putative targets related with liver cancer through network pharmacology and bioinformatics analysis. Enrichment analysis showed that G-Rk1 and G-Rg5 may induce apoptosis of liver cancer cells by targeting the critical proteins involved in MAPK and NF-κB signal pathways. Then, we proved that G-Rk1 and G-Rg5 can significantly induce endogenous apoptotic cell death of MHCC-97H cells, which was activated by caspase-9, but not caspase-8. Meanwhile, the levels of anti-apoptotic proteins decreased in different degrees. Moreover, G-Rk1 and G-Rg5 strongly inhibited the viability of liver cancer cells, but exhibited moderate cytotoxic activity in normal tissue cells, which demonstrated the anti-tumor effect of G-Rk1 and G-Rg5 in human liver cancer cells.

## Data Availability

The data presented in this study are available in supplementary material.

## Supplementary Materials

The following are available online, Figure S1: The ginsenoside-target-pathway network analysis of the overlapped genes between hepatocellular carcinoma associated genes and G-Rk1/G-Rg5 target proteins, Table S1: The network topology analysis of the overlapped genes between hepatocellular carcinoma related genes and G-Rk1/G-Rg5 target proteins.

## Sample Availability

Samples of the compounds are not available from the authors.
