# Supplementary material for: The Anti-Tumor Effect and Underlying Apoptotic Mechanism of Ginsenoside Rk1 and Rg5 in Human Liver Cancer Cells
Source: Molecules. 2021 Jun 27;26(13):3926. doi: 10.3390/molecules26133926 (PMC8271777; doi:10.3390/molecules26133926)
Supplement: Supplementary file 1 [file molecules-26-03926-s001.zip › molecules-1233741-SI.pdf]

Supplementary materials

Table S1. The network topology analysis of the overlapped genes between hepatocellular carcinoma related genes and G-Rk1/G-Rg5 target proteins. Three topological indicators include degree of distribution, betweenness centrality, and closeness centrality.

Gene	Degree	Betweenness Centrality	Closeness Centrality
EGFR	197	0.1628901	0.4675723
ESR2	180	0.16551558	0.4129257
HDAC1	108	0.09374842	0.41614665
STAT3	108	0.09763135	0.4279984
HSP90AA1	91	0.04011796	0.41340566
RELA	81	0.0573418	0.40771876
ESR1	73	0.06442078	0.4496418
MAPK14	73	0.05813376	0.38422758
HDAC2	46	0.01026334	0.35626043
MAPK8	44	0.0314783	0.37478047
CDK1	43	0.02707577	0.37877174
PPARG	40	0.01685445	0.36933195
AURKA	34	0.01299936	0.35626043
AURKB	33	0.01242833	0.33755141
DNMT1	29	0.00339268	0.35272727
MTOR	28	0.0162206	0.35483871
PIK3CA	27	0.01041758	0.34013389
PPARA	16	0.00533813	0.34530744
ALDH2	14	0.00795612	0.27663988
CYP17A1	14	0.00721384	0.33054523
MAP2K1	12	6.42E-04	0.31642942
HDAC3	11	0.0028698	0.29980332
VEGFA	8	0.00299667	0.28628924
PIK3CD	7	0.00420448	0.30039414
FGF2	6	0.00394916	0.26568725
MMP1	5	5.06E-04	0.25992692
PIK3CB	5	8.00E-04	0.29589573
NOS2	5	4.54E-04	0.3080254
F2R	4	0.00375363	0.26863041
PIK3CG	4	0.00208593	0.26562111
CD274	3	0.00187441	0.21128713
CA2	3	0.00188872	0.24233477
TYMS	3	8.31E-05	0.27599586
IL2	3	9.51E-06	0.2538059
ABCB1	3	4.97E-05	0.25410812
ACE	2	6.46E-06	0.25923226
CA9	0	0	0
MME	0	0	0
LGALS9	0	0	0
HPSE	0	0	0

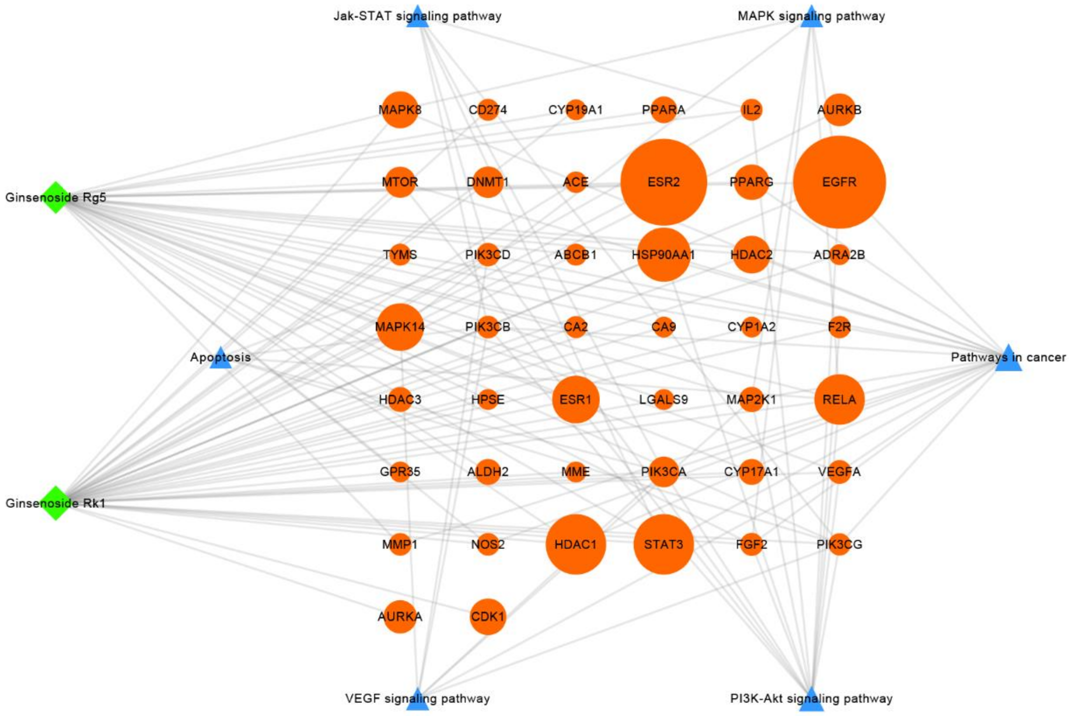

Figure S1. The ginsenoside-target-pathway network analysis of the overlapped genes between hepatocellular carcinoma associated genes and G-Rk1/G-Rg5 target proteins. The size of node corresponds to the degree of genes in human genome network as background.
